# Bright Single-Photon Emitting Diodes Based on the Silicon-Vacancy Center in AlN/Diamond Heterostructures

**DOI:** 10.3390/nano10020361

**Published:** 2020-02-19

**Authors:** Igor A. Khramtsov, Dmitry Yu. Fedyanin

**Affiliations:** Laboratory of Nanooptics and Plasmonics, Moscow Institute of Physics and Technology, Dolgoprudny 141700, Russia; khramtsov@phystech.edu

**Keywords:** color centers in diamond, single-photon source, single-photon electroluminescence, SiV centers, AlN/diamond heterojunction, interface

## Abstract

Practical implementation of many quantum information and sensing technologies relies on the ability to efficiently generate and manipulate single-photon photons under ambient conditions. Color centers in diamond, such as the silicon-vacancy (SiV) center, have recently emerged as extremely attractive single-photon emitters for room temperature applications. However, diamond is a material at the interface between insulators and semiconductors. Therefore, it is extremely difficult to excite color centers electrically and consequently develop bright and efficient electrically driven single-photon sources. Here, using a comprehensive theoretical approach, we propose and numerically demonstrate a concept of a single-photon emitting diode (SPED) based on a SiV center in a nanoscale AlN/diamond heterojunction device. We find that in spite of the high potential barrier for electrons in AlN at the AlN/diamond heterojunction, under forward bias, electrons can be efficiently injected from AlN into the i-type diamond region of the n-AlN/i-diamond/p-diamond heterostructure, which ensures bright single-photon electroluminescence (SPEL) of the SiV center located in the i-type diamond region. The maximum SPEL rate is more than five times higher than what can be achieved in SPEDs based on diamond p-i-n diodes. Despite the high density of defects at the AlN/diamond interface, the SPEL rate can reach about 4 Mcps, which coincides with the limit imposed by the quantum efficiency and the lifetime of the shelving state of the SiV center. These findings provide new insights into the development of bright room-temperature electrically driven single-photon sources for quantum information technologies and, we believe, stimulate further research in this area.

## 1. Introduction

**Figure 1:**
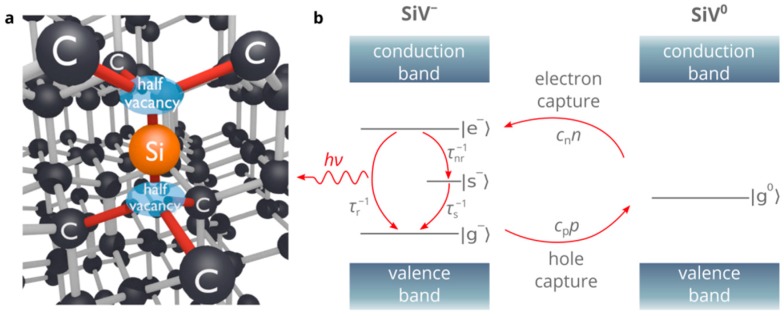
(**a**) Ball-and-stick model of the silicon-vacancy (SiV) center in diamond. (**b**) Diagram of the single-photon electroluminescence process of the SiV center in diamond after Fedyanin and Agio [9]. |g^−^>, |e^−^> and |s^−^> represent the ground, excited and shelving states of the negatively charged SiV center and |g^0^> indicates the ground state of the neutral SiV center. The transitions among the states are shown by arrows and the transition rates are indicated next to them. The notations are the same as in Equation (1).

**Figure 2:**
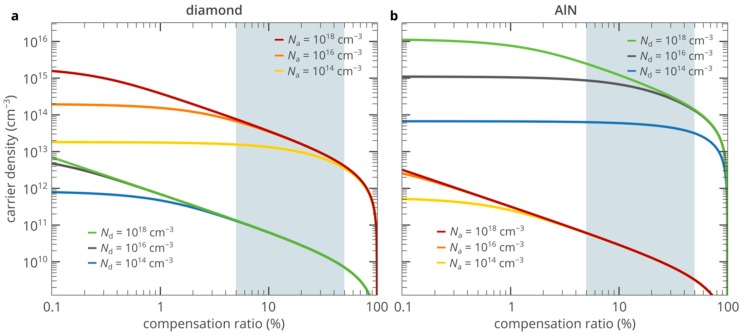
Dependences of the densities of free electrons and holes in the n-type and p-type materials on the donor and acceptor compensation ratios, respectively, for diamond (**a**) and AlN (**b**) at room temperature. Vertical grey stripes show the range of typical compensation ratios in n-type diamond and p-type AlN. The activation energies of donors and acceptors in AlN are 0.25 eV [15] and 0.63 eV [16], respectively. The activation energies of donors and acceptors in diamond are 0.57 eV [17] and 0.365 eV [18], respectively.

Practical implementation of many quantum information and sensing technologies relies on the ability to efficiently produce single photons in a scalable on-chip platform. In this regard, the development of electrically driven single-photon sources (SPSs) is crucially important since this is the only approach that allows placing a large number of different nano- and microscale SPSs on the same chip and trigger them independently using a low voltage battery [1]. In addition, electrically driven SPSs provide higher energy efficiency and better control of the single-photon emission than optically driven SPSs. At present, the most versatile platform for electrical SPSs is based on epitaxial quantum dots [2]. However, the operation of these emitters under electrical excitation is limited to cryogenic temperatures, which is not acceptable for many practical applications. On the other hand, optically driven solid-state SPSs based on color centers in diamond and related wide-bandgap semiconductor materials have demonstrated outstanding performance at room and even higher temperatures [3,4]. For example, the silicon-vacancy (SiV) center in diamond (Figure 1a) is among the most attractive room-temperature single-photon emitters. It is known for polarized emission at a wavelength of about 738 nm from the single negatively charged state, which features a narrow spectrum, large Debye-Waller factor at room and even high temperatures [5,6,7,8]. However, efficient electrical excitation of the SiV centers, as well as other color centers in diamond, is very challenging due to the inability to create a high density of free carriers, which is essential for bright single-photon electroluminescence [9,10] (Figure 1b). The reason for this is that wide-bandgap semiconductors, such as diamond, are the materials at the interface between semiconductors and insulators. The activation energy of donors and acceptors in these materials is very high, which, combined with a nonzero compensation of donors (acceptors) by acceptor-type (donor-type) impurities and defects, results in a very low density of free carriers, which can be up to ten orders of magnitude lower than the doping density. In particular, the density of free electrons in n-type diamond is limited to about 10^11^ cm^−3^ [10,11,12,13], which is much lower than the typical density of holes in p-type diamond (>10^14^ cm^−3^) (Figure 2a). In turn, the single-photon electroluminescence (SPEL) rate of a single color center in the steady state is given by [14]

(1) $$R_{\mathrm{SPEL}}=\frac{1}{1+\frac{\tau_{r}}{\tau_{\mathrm{nr}}}}\times \frac{1}{\frac{1}{c_{n}n}+\frac{1}{c_{p}p}+\tau_{r}\frac{\tau_{\mathrm{nr}}+\tau_{s}}{\tau_{\mathrm{nr}}+\tau_{r}}},$$

where *τ*_r_ and *τ*_nr_ are the radiative and non-radiative lifetimes of the excited state, *τ*_s_ is the lifetime of the shelving state, *n* and *p* are the densities of electrons and holes in the vicinity of the color center and *c*_n_ and *c*_p_ are the electron and hole capture rate constants by the color center (see Figure 1b). Equation (1) clearly shows that if the density of electrons is much lower than the density of holes, the SPEL rate is limited by *c*_n_*n*, even if the density of holes is very large and the radiative lifetime of the excited state is negligibly short. Since the electron capture rate constant for the SiV and NV centers in diamond is of the order of 10^−8^ cm^3^s^−1^ [9], the SPEL rate is of the order of less than 1 kcps.

Recent theoretical findings show that it is possible to overcome the limitation on the SPEL rate imposed by the doping of n-type diamond using the superinjection effect in homojunction diamond diodes [10,11,19]. By exploiting this effect, one can inject up to four orders of magnitude more electrons into the i-region of the diamond p-i-n diode than the doping of the n-type region allows. However, the maximum SPEL rate at 100% quantum efficiency of the color center is limited by ~3 × 10^6^ cps, which is still lower than epitaxial quantum dots can produce at low temperatures. In addition, this approach requires a relatively large size of the i-region of the p-i-n diode (~10 μm) and therefore is difficult to be employed in nanoscale devices and components. Thus, for practical applications, it is critically important to develop new schemes of electrical excitation of color centers compatible with nanoscale optoelectronic circuits.

In this work, we show for the first time that silicon vacancies and other color centers in diamond can be efficiently excited electrically using AlN/diamond heterojunctions. Using a comprehensive theoretical and numerical approach, we demonstrate that the SPEL rate of the SiV center in the i-type region of the n-AlN/i-diamond/p-diamond diode can exceed 3 Mcps, which is five times higher than what can be achieved in diamond p-i-n diodes. We find that despite the high potential barrier of 0.9 eV at the AlN/diamond heterojunction, electrons can tunnel from n-type AlN to the intrinsic diamond layer, thereby creating a high density of electrons in the i-region of the n-AlN/i-diamond/p-diamond heterostructure. At the same time, holes are injected into the i-type diamond region from the p-type diamond region. Our 2D self-consistent numerical simulations demonstrate that a high SPEL rate can be achieved in a truly nanoscale device in spite of the defects at the AlN/diamond interface.

## 2. Results and Discussion

**Figure 3:**
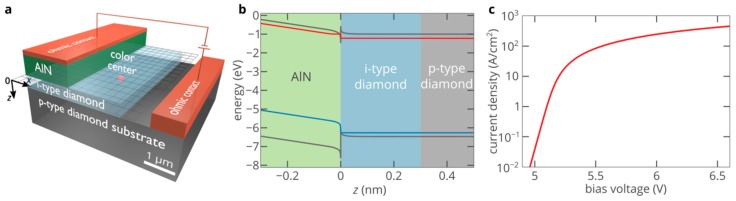
(**a**) Schematic of the AlN/diamond single-photon emitting diode (SPED) with a color center in i-type diamond region. (**b**) Energy band diagram of the SPED shown in panel a in the vicinity of the AlN/diamond heterojunction at a forward bias voltage of 6.5 V. The grey lines show the conduction and valence bands, the red line indicates the quasi-Fermi level for electrons and the blue line shows the quasi-Fermi level for holes. (**c**) Dependence of the current density at the top n-type contact on the bias voltage for the SPED shown in panel a.

Figure 3a shows the n-type AlN/i-type diamond/p-type diamond heterojunction single-photon emitting diode (SPED) based on the SiV center in the i-type diamond region of the device. AlN is doped with Si at a density of 10^18^ cm^−3^. The donor compensation ratio is equal to 10% [20]. The doping density of the p-type diamond substrate is 10^18^ cm^−3^ at a donor compensation ratio of 1%, which is typical for p-type diamond samples [18]. Free-carrier effective masses, electron and hole mobilities and other material parameters of diamond and AlN important for the operation of the SPED can be found in References [10,21]. The AlN layer plays a role of electron injection layer. Aluminum nitride, as well as diamond, is a wide-bandgap semiconductor and, therefore, also suffers from the lack of free carriers. However, fortunately, almost all wide-bandgap semiconductor materials feature a doping asymmetry. In other words, the activation energy of acceptors is typically significantly lower than the activation energy of donors or vice versa [22]. Thus, while it is extremely difficult to dope diamond n-type, it is relatively easy to create a density of free electrons of the order of 10^15^ cm^−3^ in n-type AlN (see Figure 2b). It was demonstrated that AlN is one of the few materials that can be directly grown on diamond [23,24,25,26,27]. However, the properties of the AlN/diamond heterojunctions were poorly understood, which did not allow to proceed to the development of high-performance optoelectronic devices based on them. Only very recent findings have provided a consistent picture of the formation of AlN/diamond heterojunctions [28,29,30].

Figure 3b shows the simulated energy band diagram of the AlN/diamond heterostructure at a forward bias voltage of 6.5 V. It is clearly seen that there is a conduction-band offset (CBO) of about Δ*E*_c_ = 0.9 eV [29], which creates a high potential barrier for electrons in AlN and prevents their efficient injection into diamond. The CBO depends on the polarity of the AlN/diamond interface: Δ*E*_c_ is lower for the C-Al polarity than for the C-N polarity by about 0.9 eV [29] so that there is almost no potential barrier for electrons in AlN at the AlN/diamond heterojunction. However, although it has been demonstrated that AlN can be fabricated and prepared so that the top surface of AlN has the Al polarity [31,32], to the best of our knowledge, the C-Al polarity has not been observed for AlN grown on diamond. Thus, here, we mostly focus on the worst case, that is, on the AlN/diamond interface with the C-N polarity. The valence-band offset (VBO) at the AlN/diamond interface is about 0.6 eV higher than the CBO (Figure 3b). Therefore, the potential barrier for holes in diamond is significantly higher than the potential barrier for electrons in AlN. Thus, the density of holes injected into AlN from diamond is much lower than the density of electrons injected into diamond from AlN and the current through the heterojunction is predominantly the electron injection current.

Although the electron current is orders of magnitude higher than the hole current, its absolute value can be very low. The high potential barrier for electrons at the AlN/diamond heterojunction casts doubt on the possibility of efficient electron injection and, consequently, on the feasibility of bright SPEL of the SiV center in the i-diamond region. The electron current density through the junction due to the over-barrier electron emission is proportional to $1/4q\upsilon_{\mathrm{th}}n{|}_{z=-0}\exp\left(-\Delta E_{c}/kT\right),$ where *υ*_th_ is the electron thermal velocity in AlN, *n*|_*z* = −0_ is the electron density in AlN and *kT* is the thermal energy. At room temperature exp(−Δ*E*_c_/*kT*)~10^−16^, hence the only mechanism of electron transport through the heterojunction is tunneling. At moderate injection levels, the electron tunneling current density can be expressed as

(2) $$J=\frac{qm_{e}^{\mathrm{AlN}}kT}{2\pi^{2}\hbar^{3}}\int_{-\infty }^{\infty }T(E)\ln\left[1+\exp\left(\frac{F_{n}{|}_{z=-0}-E}{kT}\right)\right]dE,$$

where *T*(*E*) is the transparency of the potential barrier, which is given by

(3) $$T(E)=\exp\left(-\frac{2}{\hbar }\int_{0}^{z_{0}}\sqrt{2m_{d}^{t}\left(E_{c}(z)-E\right)}dz\right).$$

In the above equations, *E*_C_(*z*) is the conduction band in diamond, *z*_0_ is the classical turning point for the potential barrier, that is, *E*_c_(*z*_0_) = *E*, *F*_n_|*_z_*_= −0_ is the quasi-Fermi level for electrons in AlN in the vicinity of the AlN/diamond heterojunction, *m*_d_^t^ is the tunneling electron mass in diamond, *m*_e_^AlN^ is the effective electron mass in AlN, *ħ* is the reduced Planck constant and *q* is the elementary charge.

**Figure 4:**
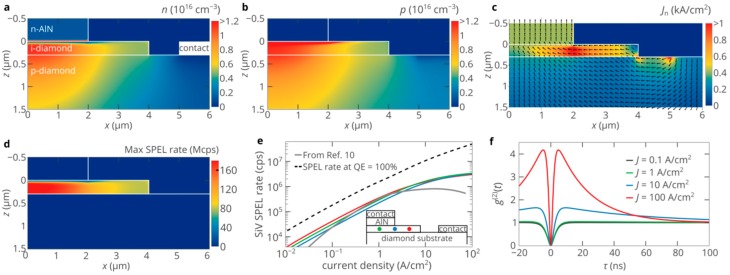
(**a**–**c**) Distributions of the electron density (**a**), hole density (b) and electron current density (**c**) in the SPED shown in Figure 3a at a bias voltage of 6.5 V. (**d**) Dependence of the single-photon electroluminescence (SPEL) rate of the SiV center on its position in the i-type diamond region of the SPED shown in Figure 3a. (**e**) Dependence of the SPEL rate of SiV center on the injection current for three different positions of the center in the i-region shown in the insert. The grey curve shows the dependence of the maximum SPEL rate of the SiV center in a diamond p-i-n diode from Reference [10]. The dashed line shows the SPEL rate at 100% quantum efficiency of the SiV center and the lifetime of the excited state of ~1 ns. (**f**) *g*^(2)^ function for the SiV center located at *x* = 1 μm, *z* = 150 nm for different pumping levels. The lifetime of the excited state is 1.2 ns, the lifetime of the shelving state is 100 ns [36] and the quantum efficiency is equal to 30% [33].

When a positive voltage is applied to the SPED, a potential well for electrons is formed in AlN near the AlN/diamond heterojunction. Under high forward bias, the density of electrons accumulated in this potential well can be as high as ~10^19^ cm^−3^. At the same time, the gradient of the electric field at the heterojunction is proportional to the accumulated charge density. Therefore, the width of the barrier for electrons decreases to a few nanometers so that electrons can tunnel to the i-type diamond region of the structure. Figure 3c shows the results of the self-consistent 2D numerical simulations of the electron and hole transport in the AlN/diamond SPED obtained using Atlas Silvaco. In spite of the very large CBO at the AlN/diamond interface, at high forward bias, the electric field at the junction exceeds 5 × 10^6^ V/cm and the potential barrier for electrons reduces to less than ~3 nm. Thus, the current density can be as high as 100 A/cm^2^, even at moderate bias voltages. Accordingly, the density of electrons injected into diamond exceeds 3 × 10^15^ cm^−3^ (Figure 4a), that is, it is even slightly higher than the electron density in the bulk of AlN. Holes from the p-type diamond substrate are also injected into the i-type diamond region under forward bias. However, the VBO at the heterojunction is higher than the CBO. Thus, holes almost do not penetrate into the AlN layer (Figure 4b).

Figure 4c shows the heatmap of the current density in the SPED. Despite that the i-type diamond region is only 300-nm-thick and the lateral dimension is much larger than the vertical one, the injected electrons flow from the AlN injection region toward the p-type contact predominantly in the i-type region along the *x*-axis. This ensures a rather homogeneous distribution of electrons and holes in the i-type diamond region of the device (Figure 4a,b). Therefore, the SPEL rate almost does not depend on the position of the SiV center in the i-region (Figure 4d), which allows to achieve bright single-photon emission not only from color centers located underneath the AlN layer (see the green dot in the insert of Figure 4e) but also from color centers in the i-type region not covered by any semiconductor or metal layer (see the red dot in the insert of Figure 4e). Such a property is beneficial for efficient extraction of photons emitted by the color center. The high density of injected carriers gives the possibility to achieve ultrahigh SPEL rate at moderate injection currents (Figure 4e). We find that the maximum SPEL rate is roughly five times higher than in diamond SPEDs based on p-i-n structures that exploit the superinjection effects [10,11]. For a single SiV center in diamond, the SPEL rate is equal to 2.9 × 10^6^ cps at a current density at the top contact of 50 A/cm^2^ and is as high as 3.9 × 10^6^ cps at 500 A/cm^2^. We should emphasize that as follows from equation (1), at high injection currents, the SPEL rate is limited only by the lifetime of the shelving state and the quantum efficiency of the SiV center, which is about 30% according to the recent experimental results [33]. If the quantum efficiency were higher and/or the lifetime of the shelving state were shorter, the SPEL rate would be considerably higher, which is indicated by the dashed line in Figure 4e.

Figure 4f shows the evolution of the second-order autocorrelation function *g*^(2)^(*τ*) of the electrically pumped SiV center in the i-type diamond region of the SPED with the injection current. In contrast to the electrically pumped NV center in diamond [34,35], the lifetime of the shelving state of the SiV center is relatively long (~100 ns [36]). Therefore, at high SPEL rates, strong photon bunching can be observed (Figure 4f), which is a clear sign of the high SPEL rate. Figure 4a,b shows that even at high injection levels, the densities of electrons and holes in the i-type diamond region are roughly the same. Therefore, the shape and characteristic times of the *g*^(2)^ function of the SiV center in the AlN/diamond SPED can be used for indirect measurements of the SPEL rate using the approach reported in References [34,35], which is very helpful, since it is typically difficult to directly measure the SPEL rate due to the poor collection efficiency due to the high refractive index of diamond, while the *g*^(2)^ function is not sensitive to the collection efficiency.

**Figure 5:**
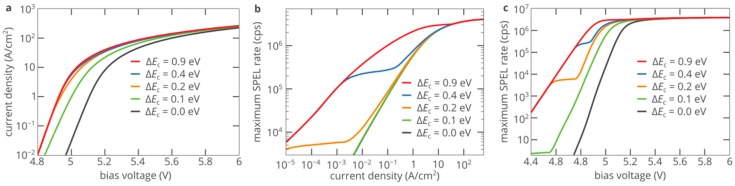
(**a**) Current-voltage characteristics of the SPED shown in Figure 3a for different conduction band offsets (CBOs) at the AlN/diamond interface. (**b**,**c**) Dependence of the SPEL rate of the SiV center on the injection current (**b**) and bias voltage (**c**) for different CBOs at the AlN/diamond interface.

The brightness of the SPED is determined by the efficiency of electron injection from the AlN layer into the diamond region. Hence, the SPEL rate of the color center increases as the CBO at the AlN/diamond interface decreases since it becomes easier for electrons in AlN to penetrate into diamond at lower potential barriers. At low CBOs, electrons are efficiently injected from AlN into diamond even without tunneling, via thermionic emission. However, it should be noted that at A bias voltage above ~5.6 V, the current-voltage characteristic does not change appreciably as the CBO decreases (Figure 5a). This proves that the efficiency of electron injection through the barrier at the heterojunction is high even at a CBO of 0.9 eV. Since the electron density in the i-type diamond region is determined by the electron injection current, at current densities above 30 A/cm^2^, the dependence of the SPEL rate on the pump current is the same for all CBOs (Figure 5b). At the same time, a clear difference between the structures with different CBOs can be observed at voltages below 5.2 V (current densities below 10 A/cm^2^) (Figure 5b,c), which is determined by the CBO at the AlN/diamond interface.

Figure 5b,c shows that the AlN/diamond heterojunction SPED based on color center in diamond demonstrates outstanding brightness, which is much higher than what can be achieved with diamond p-i-n structures. However, the lattice constant of AlN is not equal to that of diamond and, therefore, it is not possible to fabricate an ideal, defect-free AlN/diamond interface. These defects can be charged and affect the transport of free carriers across the heterojunction and, consequently, the dependence of the SPEL rate on the bias voltage. We performed self-consistent simulations of the SPED and found that this effect is negligibly small and does not influence the dependence of the SPEL rate on the injection current. At the same time, electrons and holes can recombine at deep-level defects at the AlN/diamond interface. However, not all defects at the interface are involved in the recombination process, only less than 10% or even 0.1% of them act as recombination centers [37,38].

**Figure 6:**
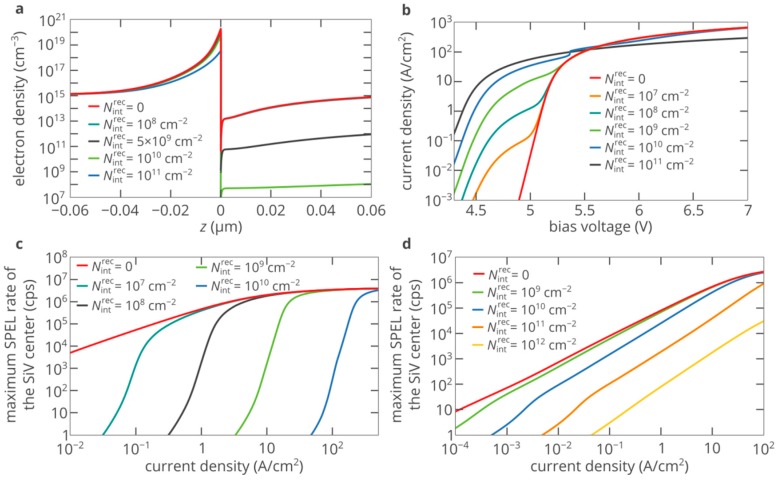
(**a**) Electron density distribution in the vicinity of the AlN/diamond heterojunction at a bias voltage of 5.3 V for different densities of recombination centers at the AlN/diamond interface. The CBO at interface is equal to 0.9 eV. (**b**) Current-voltage characteristic of the SPED for different densities of recombination centers at AlN/diamond interface. The CBO is 0.9 eV. (**c**,**d**) Dependence of the maximum SPEL rate of the SiV center in the i-type region of the AlN/diamond SPED on the pump current density for different densities of recombination centers at the AlN/diamond interface at a CBO of 0.9 eV (panel c) and 0.4 eV (panel d).

Unlike defects in the bulk of AlN and diamond located near the interface, the interface defects can capture electrons and holes from both semiconductors, which is especially critical for forward-biased p-n heterojunctions. Therefore, models that interpret interface defects as an ultrathin layer filled with defects give fundamentally wrong results, since in these models, defects in one semiconductor cannot capture free carriers from the other semiconductor. Under forward bias, electrons are accumulated in AlN in the vicinity of the AlN/diamond interface (Figure 6a), while holes are accumulated in diamond in the vicinity of the AlN/diamond interface. If there are no defects at the interface, these accumulated carriers do not directly interact with each other. The presence of defects changes this. Interface defects capture electrons and holes from both materials. This recombination contributes to the current through the devices. At a bias voltage above ~4.5 V, the recombination rate at the interface can be given by

(4) $$U_{\mathrm{int}}=\frac{N_{\mathrm{int}}^{\mathrm{rec}}\left(c_{n}^{\mathrm{AlN}}n{|}_{z=-0}+c_{n}^{d}n{|}_{z=+0}\right)\left(c_{p}^{\mathrm{AlN}}p{|}_{z=-0}+c_{p}^{d}p{|}_{z=+0}\right)}{c_{n}^{\mathrm{AlN}}n{|}_{z=-0}+c_{n}^{d}n{|}_{z=+0}+c_{p}^{\mathrm{AlN}}p{|}_{z=-0}+c_{p}^{d}p{|}_{z=+0}},$$

where *N*^int^_rec_ is the density of recombination centers at the AlN/diamond interface, *c*_n_^AlN^ and *c*_n_^d^ are the electron capture constants for electrons in AlN and diamond, respectively, and *c*_p_^AlN^ and *c*_p_^d^ are the hole capture constants for holes in AlN and diamond. Accordingly, the contribution of the interface recombination to the current through the device is equal to *qU*_int_, that is, *J*_n_|_z = −0_ = *J*_n_|_z = +0_+*qU*_int_ and *J*_p_|_z = −0_ = *J*_p_|_z = +0_-*qU*_int_. The interface recombination depletes the density of electrons in AlN in the vicinity of the heterojunction *n*|*_z_*_= −0_. Consequently, the electric field at the heterojunction decreases as *N*^int^_rec_ increases and the width of the potential barrier for electrons increases, which prevents electrons in AlN from injection into diamond.

Figure 6a shows the results of the self-consistent 1D numerical simulations of the n-AlN/i-diamond/p-diamond SPED with the thicknesses of the AlN and i-diamond layers as in Figure 3 and the thickness of the p-diamond layer of 2 μm at different densities of the recombination centers at the AlN/diamond interface. The CBO at the AlN/diamond interface is equal to 0.9 eV. At a fixed bias voltage, the density of electrons in AlN near the heterojunction decreases as *N*^int^_rec_ increases. As a result, the efficiency of electron injection into the i-type diamond layer drops dramatically, since the tunneling current decreases exponentially with the barrier thickness (see Equations (2) and (3)). The density of recombination centers at the interface of less than 10^10^ cm^−2^ almost does not affect the electron injection properties of the heterojunction at *V* = 5.3 V. At the same time, the density of recombination centers of greater than 10^11^ cm^−2^ suppresses electron injection. At higher voltages, more electrons are accumulated in the potential well near the AlN/diamond interface, which facilitates the electron injection. Accordingly, the impact of the interface defects is weaker, which can be seen in the current-voltage characteristics, which almost coincide at very high injection currents (Figure 6b).

Figure 6a shows that for efficient electron injection the density of electrons accumulated in the AlN in the vicinity of the AlN/diamond interface should be of the order of *n*_threshold_ = 10^20^ cm^−3^ at a CBO of 0.9 eV. At the same time, using Equation (4) and the boundary conditions for electrons and holes at the heterojunction, we can easily obtain that such a density corresponds to recombination rate at the interface defects of the order of *c*_n_^AlN^*n*_threshold_*N*_int_^rec^, which corresponds to the current density due to surface recombination of

(5) $$J_{\mathrm{threshold}}=qc_{n}^{\mathrm{AlN}}n_{\mathrm{threshold}}N_{\mathrm{int}}^{\mathrm{rec}}.$$

This equation gives a good estimation of the threshold current at which efficient injection of electrons from the n-type AlN layer into the i-type diamond layer begins. Thus, at current densities above *J*_threshold_, defects at the AlN/diamond interface do not pose a problem for efficient electron injection and, consequently, bright SPEL of the color center in the i-type diamond region of the SPED is possible. At a CBO of 0.9 eV at the AlN/diamond interface, we obtain that

(6) $$J_{\mathrm{threshold}}=16\frac{N_{\mathrm{int}}^{\mathrm{rec}}}{10^{9}{\mathrm{cm}}^{-2}}{A/\mathrm{cm}}^{2},$$

which is in excellent agreement with the precise numerical simulations of the SPEL rate of the SPED shown in Figure 6c. At a CBO of 0.4 eV at the AlN/diamond interface, the threshold electron density is about 10^19^ cm^−3^. Accordingly, the threshold current is an order of magnitude lower than at a CBO of 0.9 eV (Figure 6d). In addition, due to the relatively low barrier height, thermionic emission significantly contributes to the electron injection current. Therefore, efficient electron injection is possible even at a density of recombination centers at the interface of the order of 10^12^ cm^−2^, which roughly corresponds to the density of interface defects of the order of 10^13^ cm^−2^.

## 3. Conclusions

We propose and numerically demonstrate the concept of a room-temperature single-photon emitting diode based on a color center in the i-type region of the n-AlN/i-diamond/p-diamond heterostructure. We find that the SPEL rate of the SiV center in such a diode can exceed 3 Mcps at a bias voltage of less than 6 V despite the relatively low quantum efficiency of the SiV center and the long lifetime of its shelving state. This value is five times higher than the maximum SPEL rate of the SiV center in SPEDs based on diamond p-i-n diodes [10,11]. The maximum SPEL rate at 100% quantum efficiency of the color center exceeds 40 Mcps, which is an order of magnitude higher than what has been predicted for color centers in p-i-n diamond diodes. We show that such a remarkably high SPEL rate can be achieved in a truly nanoscale device, which is required for building nano-optoelectronic components for practical quantum technologies.

We also analyze the impact of defects at the AlN/diamond interface on the performance of the heterostructure SPED. We find that at a CBO of 0.9 eV, the density of recombination centers at the AlN/diamond interface of less than 10^10^ cm^−2^ does not affect the performance of the SPED at high bias voltages, while the density of recombination centers higher than 2 × 10^11^ cm^−2^ completely suppresses electron injection from AlN to diamond and limits the brightness of the SPED to zero. As the CBO at the hetero-interface decreases, the impact of defects decreases. At a CBO of 0.4 eV, bright SPEL can be achieved even at a density of recombination centers higher than 10^12^ cm^−2^. The CBO at the AlN/diamond interface might be decreased for both interface polarities using oxygen passivation [23,39,40], which should reduce the impact of interface defects on the SPED performance. However, such experimental studies are yet to be performed. We emphasize that only a small portion of defects participate in the recombination process and therefore the density of interface defects is 10–1000 times higher than the density of recombination centers. Thus, although it is not clear yet to what extent the quality of the AlN/diamond heterostructures can be improved, we demonstrate that the density of interface defects of less than 10^11^–10^12^ cm^−2^ cannot affect the performance of the AlN/diamond SPED, which holds promise for the development of bright room-temperature electrically driven single-photon sources and forms a solid foundation for further research in this area.
